# STEM the bullying: An empirical investigation of abusive supervision in academic science

**DOI:** 10.1016/j.eclinm.2021.101121

**Published:** 2021-09-08

**Authors:** Sherry E. Moss, Morteza Mahmoudi

**Affiliations:** a School of Business, Wake Forest University, NC, USA; b Department of Radiology and Precision Health Program, Michigan State University, MI, USA

## Abstract

**Background:**

Academic bullying is a topic of significant interest of late, with high profile cases featured in scientific journals. Our aim is to document the nature and extent of academic bullying behaviors, examining who are the primary targets and perpetrators as well as the responses to and outcomes of bullying.

**Methods:**

We developed a cross-sectional global survey aimed primarily at those in academic science institutions. The survey was administered *via* Qualtrics and data were collected (between November 2019 and July 2021) from 2006 individuals whose participation was solicited through various means including advertisements in Science and Nature magazines and the American Chemical Society.

**Findings:**

Among the 2006 survey participants, the majority of targets were graduate students or postdocs. An overwhelming proportion of participants reported either experiencing (84%) or witnessing (59%) abusive supervision, or both (49%). While a majority of perpetrators were male, they were proportionately no more likely to abuse than females. Perpetrators were more likely from the highest-ranked institutions and they were most likely PIs. Females were more likely to report being bullied but their scores on the Tepper abusive supervision scale and the contextual behavior checklist we developed were not greater than male targets. Male targets actually reported higher levels of certain bullying behaviors. While international scholars were no more likely to report being bullied, the severity of the behaviors they reported was significantly greater. Targets (64%) were most likely to use avoidant tactics (not reporting and relying on family/friends for support) in response to bullying due to fear of retaliation (61%). The small percentage that did report the abuse (29%) overwhelmingly reported unfair and biased (58%) outcomes. Additional qualitative analysis of open-ended comments revealed similar patterns. We also noticed that the COVID-19 pandemic has exacerbated academic bullying and changed the patterns of behaviors possibly due to the remote nature of interactions. Open-ended responses from targets are analyzed with examples provided.

**Interpretation:**

Our results elucidated the various forms of abuse, the most likely perpetrators and targets, as well as the typical reactions of targets and witnesses. We investigated the results of targets’ actions following chronic bullying. Our findings highlight the domain, extent, and dynamics of academic bullying to hopefully motivate the scientific community to take action.

**Research in context**

**Evidence before this study**

While there is significant anecdotal and empirical evidence to suggest that academia is “rife” with bullying from many different sources, there has been less specific focus on the hierarchical nature of bullying. Additionally, studies tend to utilize only one measure of bullying such as a single item or a general behavioral checklist. The most often-used checklists are general measures of “negative acts” in the workplace that could be perpetrated by many different actors.

**Added value of this study**

Our study is intended to be a comprehensive evaluation of hierarchical bullying, using three different measures: Single item, the generalizable Tepper (2000) scale for abusive supervision, and a checklist of behaviors that we created specifically to represent the context of academic science. In addition, we examine not only the most likely perpetrators and victims, but also the perspective of witnesses and the responses of both targets and witnesses following abusive supervision. We present both quantitative and qualitative data that fully capture the experience of bullying from academic superiors.

**Implications of all the available evidence**

This study suggests that academic science has a significant problem with abusive supervision, emanating from academic superiors (*e.g.* principal investigators) and directed toward academic subordinates (*e.g.* graduate students and post-docs). Evidence suggests that bullies are more likely to come from the highest ranked institutions. The way bullying was measured produced differences between male and female targets. International scholars indicated a higher level of contextual abuse than domestic scholars. Both witnesses and targets generally did not report bullying due to fear of retaliation. Those that did report primarily reported unfair and biased outcomes. The COVID-19 pandemic has exacerbated academic incivility and changed the patterns of behaviors.

Alt-text: Unlabelled box

## 1 Introduction

“Academic bullying” has become a topic of great interest in the last several years [1], [2], [3], [4], [5], [6]. While there has been some systematic study of this phenomenon, our goal is to provide a specific definition and operationalization and an empirical narrative of the extent of abusive behaviors in academic science. At the same time, “abusive supervision” has been a topic of interest for several decades in the organizational literature [7,8]. Defined as subordinates’ perceptions of the extent to which their supervisor engages in a sustained display of hostile verbal and nonverbal behaviors, excluding physical contact [7], typical behaviors include ridicule and various forms of verbal abuse such as blaming, put-downs, angry outbursts, and name-calling [7]. It also involves isolating targets, giving them “the silent treatment,” and invading their privacy. The literature on abusive supervision has mainly focused on the consequences of such behavior including lower job satisfaction, anxiety, depression, emotional exhaustion, perceived injustice, workplace deviance, lowered performance, and turnover [8]. In this paper, we apply the knowledge from the organizational literature to the specific context of academic science. We examine the extent of abusive supervision in this context and extend the work to include contextual abusive behaviors that are specific to the process of scientific inquiry. We seek to understand who are the most likely targets, the most likely perpetrators, and the responses and consequences of these behaviors. Our hope is that our findings illuminate the prevalence of academic bullying and motivate the scientific community to create resources to address it.

Which bullying behaviors occur most frequently in academic science? We recommend that the study of what has colloquially been termed “bullying” in academic science rely, in part, on the science of abusive supervision established in the organizational literature. Tepper [7] developed the domain of “abusive supervision” by drawing on the domestic violence literature. Aside from physical abuse, the specific behaviors demonstrated by domestic abusers and workplace abusers are very similar. These can be verbal (*e.g.*, name calling, put-downs, blaming) and non-verbal (*e.g.*, silent treatment, isolation) and present themselves in a variety of contexts such as healthcare [9] and hospitality [10], [11], [12] (See Table 1 for scale items), though Tepper developed his scale as a measure of abusive supervision in organizations in general. There is now significant anecdotal [13] and empirical [6] evidence of such behaviors in academic science. At the same time, there is behavior specific to academic science that must also be captured in any systemic study of academic bullying. This behavior includes abusing authorship or violating intellectual property rights [14]; threatening to cancel funding, positions, or visas [15]; and damaging budding scientists’ reputations through bad recommendations or speaking negatively about them to others [16]. We sought to evaluate the effectiveness of Tepper's measure to understand the extent of abusive supervision in academic science. And we also sought to develop an additional measure of contextual behaviors to help understand specific abuses unique to the lab and educational or scientific institutions. Thus, we created an inventory of behaviors, based on context-specific anecdotal narratives, to specifically assess academic bullying. We report on the extent of bullying using each method and compare the efficacy of Tepper's scale to our contextual checklist. By combining the organizational definition of abusive supervision with the contextual checklist, we define academic bullying as *sustained hostile behavior from one's academic superior including, but not limited to, ridiculing, threatening, blaming, invasion of privacy, putdowns in front of others as well as interference with matriculation and career progress including removing funding, writing falsely negative recommendation letters, taking credit for others’ work and threatening to cancel visa or fellowships.”* Our research question is: Which bullying behaviors occur most frequently in academic science? In addition to assessing this question, we also develop and test a series of hypotheses, based on the organizational literature, about the most likely targets and perpetrators of bullying as well as their likely responses and the consequences of their actions.

**Table 1:** Tepper scale items and means for targets*My supervisor….

Tepper scale item	Target mean	Std. deviation
Ridicules me.	3.26	1.37
Reminds me of my past failures or mistakes.	3.24	1.42
Tells me my thoughts or feelings are stupid.	2.88	1.45
Tells me I'm incompetent.	2.95	1.49
Expresses anger at me when he/she is mad for another reason.	3.42	1.49
Makes negative comments about me to others.	3.73	1.39
Puts me down in front of others.	3.43	1.41
Blames me to save him/herself embarrassment.	3.28	1.56
Gives me the silent treatment.	3.14	1.61
Does not allow me to interact with my coworkers.	2.58	1.60
Doesn't give me credit for my work.	3.41	1.54
Invades my privacy.	2.69	1.59
Doesn't give me credit for jobs requiring a lot of effort.	3.69	1.45
Breaks promises he/she makes.	3.50	1.58
Lies to me.	3.46	1.58
Overall mean (Scale 1–5)	3.23	.89

*N = 1131 (note that some who indicated that they had been bullied from the single item did not complete all survey items).

Who are the perpetrators? The literature on abusive supervision in organizations is clearly based on differences in power between perpetrators and targets. Abusive supervision, by definition, refers to perceptions that one in a “supervisory” status is perceived as perpetrating harmful acts toward another in an inferior position.

The literature on abusive supervision in organizations further identifies three categories of antecedents: self-regulation impairment, identity threat, and social learning [8]. First, the literature on leadership reveals that leadership styles are learned by followers and often repeated [17,18]. This so-called trickle-down effect implies that the behaviors of abusive leaders are emulated by followers [18] and are actually more likely to be learned and passed down than positive behaviors. Leaders who “grew up” with an abusive principal investigator (PI) or department chair are more likely to assume that their own followers must also “pay the dues” of working in the tough field of academic science [19,20]. Graduate students or post-docs who would give up just about anything to work with famous scientists from top-tier institutions may be willing to put up with abuse just to work in a particular scientist's lab, regardless of that scientist's reputation for bullying [21]. The social learning effect may result in these budding scientists passing along the same abusive behaviors, when they are in the position to do so.

Second, research suggests that those in positions of authority may feel threatened by their own superiors or even subordinates, and these perceived threats often precipitate abuse. In the case of academic science, threats from above may include pressure to publish [22] or obtain grants [23], while threats from below may include incompetent or otherwise dysfunctional subordinates who make the leader appear or feel incompetent [9]. Threats may also emanate from within. Those with “dark triad” personality traits (*i.e.*, Machiavellianism, narcissism, and psychopathy) are more likely to bully because they feel justified and have little empathy for others [19,24,25].

Finally, exhausted, over-worked scientists may experience a depletion of personal or psychological resources [26] and react harshly to provocation from subordinates through conflict or poor performance [9]. Each of these antecedents suggests that power differentials between PIs and graduate students or postdocs, for example, exacerbate the likelihood that those in positions of authority may unleash their wrath toward those in less powerful positions [15,16,27]. The unique aspects of science, which require focused work on a series of studies or experiments that may eventually pay off in terms of scientific value, increases the likelihood that early-career scientists (*e.g.,* graduate students or post-docs) will feel “stuck” in their labs, perceiving little opportunity to change their circumstances without losing months or even years of work [15]. This is consistent with evidence in organizational research that the lack of “perceived alternatives” is a powerful predictor of a target being willing to stay with an abuser [7]. We hypothesize that abusers will typically be in positions of greater power along a number of dimensions such as gender, relative position, and institutional rank. Therefore, our first hypothesis is “*Perpetrators are hierarchically superior to targets.”*

As discussed above, it is highly probable that power differentials are exacerbated in prestigious institutions due to the high demand for positions in the labs of highly successful scientists [28]. In short, it is easier to “get away with” abusive behavior when lab members believe themselves to be fortunate to even have their positions. Therefore, our second hypothesis is” *Perpetrators are more likely to work in highly ranked institutions.”*

Although women have historically made up a considerable proportion of the STEM and health workforce [29], there is significant evidence that women have difficulty advancing in STEM careers [30] and are twice as likely as men to leave STEM careers [31]. This may be due, at least in part, to gender inequities at higher academic ranks (*e.g.*, full professor and chair positions), greater likelihood of bullying by the male majority and even discrepancies in award money and/or prestige [32,33]. Thus, our third hypothesis is ”*Perpetrators of bullying are disproportionately male.”*

Who are the targets? Corresponding to our hypotheses that perpetrators are more powerful, we argue that targets of academic bullying are less powerful. There is substantial anecdotal [23,34] and empirical evidence that minorities and women are more likely targets of abusive supervision in general. Our fourth hypothesis is ”*Targets of bullying are disproportionately female.”*

In addition, the research on abusive supervision and bullying in academia suggests that individuals who are dissimilar to the abuser are more likely to be targeted [9,19,35]. Linguistic and cultural barriers, together with visa issues and less family support make international scholars more vulnerable to bullying. Thus, our fifth hypothesis is “*Targets of bullying are disproportionally international scholars.”*

What are the consequences of bullying? Here we develop three hypotheses: The first concerns the most likely actions taken by targets (and witnesses) after a bullying incident. The second proposes a rationale for these actions, and the third explains the most likely results of targets’ allegations of bullying.

Most adults must maintain functional relationships with various disagreeable individuals, who may be relatives, roommates, colleagues, or bosses. Bullying (*a.k.a.* abusive supervision) creates an unwanted relationship, and targets are left to determine how they will deal with inevitable interactions with the perpetrator.

The natural human reaction to a perceived threat or attack is either a “fight or flight” response [36]. In challenging relationships, fighting typically means taking a direct approach such as discussing relationship problems, communicating expectations and boundaries or questioning relational injustices [9], either directly with the perpetrator or the institution. Flight responses are typically attempts to escape from noxious stimuli before they occur [37]. In the case of bullying, this might take the form of avoiding the threat (*i.e.,* bully) through interpersonal distancing [38].

In an organizational study [39], individuals who perceived their supervisors as abusive were significantly more likely to engage in avoidant behaviors than direct confrontation, even though the latter made them feel better (*i.e.,* less anxious). Accordingly, our sixth hypothesis is that “*Targets and witnesses will more likely use “flight” than “fight” tactics in response to bullying.”*

To further explain the likelihood of this response, Von Elm et al. [39] suggest that while directly confronting the perpetrator might be more efficacious, it is unlikely for several reasons. First, targets may be concerned about the personal costs associated with speaking out. Anecdotal evidence suggests that there is significant fear that the perpetrator will make the target's life even harder [6]. Our seventh hypothesis is “*Fear of retaliation is the primary reason for avoidance tactics.”*

Finally, through anecdotes collected from targets and witnesses, as well as documented accounts of high-profile bullying cases, we suspect that when targets do decide to report bullying, institutions rarely offer fair and unbiased responses. Thus, our eighth hypothesis is “*Targets and witnesses perceive that institutional responses to reports of bullying by targets are inadequate.”*

What is the impact of COVID-19 on bullying? Since some of our data were collected during the COVID-19 pandemic, we had the opportunity to include survey questions to assess whether bullying had increased or decreased during the pandemic. Of our 2006 participants, 206 provided responses to the COVID question. In order to obtain more data regarding possible changes in patterns of academic bullying, we conducted a separate survey and received 191 responses (see the Methods section for full survey details). Our ninth hypothesis is *The COVID-19 pandemic has exacerbated abusive behavior and changed its frequency and patterns.”*

## 2 Methods

### 2.1 Survey

Full information about the IRB approval and consent and declaration of informed consent to use the data from the participants is provided in the survey details in the Appendix file. Briefly, we provided the information about the study, IRB approval, and the use of anonymous data on the first page of the survey. Participants that agreed to proceed, indicated that they were at least 18 years old and that they agreed to participate in the research project. The outcomes of our study were reported according to the Strengthening Reporting of Observational Studies in Epidemiology (STROBE) guidelines [40].

### 2.2 Main study

Data were collected from 2006 individuals whose participation was solicited through various means including advertisements in *Science* and *Nature* magazines (through an advertorial piece [41] and third-party emails) and the American Chemical Society (through their online panel advertisement and third-party emails). Participants were 65% female, 66.5% white (11.8% Asian, 6% Hispanic, 7% Middle eastern, 2.8% mixed race, 1.8% East Indian, and 1.3% Black), and 60% were residents of the country in which they studied or worked when experiencing/witnessing bullying. The participants were primarily postdocs (22.8%) and graduate students (21.6%) with 17% junior faculty, 13% senior faculty, and 21% “other” (indicating that staff members or other professionals were respondents).

The majority of participants came from the fields of life science (19%), social sciences (13.8%), chemistry (8.8%), engineering (8.7%), neuroscience (7.4%), molecular biology (7.6%) and physical science (5.4%). Other fields were represented including biotech/pharma, clinical science, genetics, cancer research, immunology, earth science and math/computational sciences (all ranging from 1.9% to 3.4%).

Forty-eight percent of the bullying reported took place in the U.S. The most represented states were Massachusetts (12.8%), California (12.1%), New York (9.1%), Texas (6.8%), and North Carolina (5.7%).

### 2.3 Measures

After giving their informed consent to participate, respondents were given a prompt that provided a definition of academic bullying: *Academic bullying is defined as sustained hostile behavior from one's academic superior including, but not limited to, ridiculing, threatening, blaming, invasion of privacy, put-downs in front of others as well as interference with matriculation and career progress including removing funding, writing falsely negative recommendation letters, taking credit for others' work and threatening to cancel visas or fellowships.* They were then asked to indicate if they had ever been the target of such behavior. Those who responded “no” were directed to the portion of the survey related to witnesses of bullying. Those who answered “yes” were directed toward questions about the perpetrator such as his/her role (*e.g.*, PI), sex, and age as well as characteristics of the institution in which the bullying took place (*e.g.*, rank). After responding to these questions, they proceeded to the section asking them about specific bullying behaviors.

We used the 15-item abusive supervision scale developed by Tepper [7] to assess generic bullying behaviors. Sample items were “my supervisor ridicules me” and “my supervisor puts me down in front of others." Participants who indicated that they perceived they had been bullied responded to these items using a 5-point Likert scale ranging from 1 (“I cannot remember him/her ever using this behavior with me”) to 5 (“He/she uses this behavior very often with me.”) Items were adjusted slightly for participants who had witnessed bullying (*e.g.*, “the perpetrator ridicules others”). The reliabilities for the Tepper scale were more than adequate: alpha of 0.87 for targets and 0.91 for witnesses.

In addition to the Tepper scale, we developed a contextual behavior checklist comprising items developed through the analysis of publicized stories and narratives of victims collected over time by the authors. We call this the Abusive Supervision in STEM Checklist (AbSuSTEM Checklist). The scale consists of 10 items (See Table 2**)**. We believe that it is more interesting to examine the specific behaviors on the checklist than to provide a global “score.” This will inform training for science leaders and enable institutions to develop specific policies.

**Table 2:** Contextual scale items and means for targets*The perpetrator….

Abusive supervision in science (contextual items)	Target%
gave me a bad/unfair recommendation.	48.0
canceled or threatened to cancel my visa.	8.9
Unnecessarily lengthened my stay in his/her lab.	33.6
Took away my funding or threatened to take away my funding.	43.1
Encouraged others to mistreat me.	53.1
Used my data in papers/patents without acknowledging my contribution.	36.5
Violated authorship contribution guidelines (if existed).	41.0
Forced me to sign away my rights.	16.0
violated my intellectual property rights.	29.3
canceled or threatened to cancel my current appointment/position.	52.1

*N = 1128 (note that not all who indicated they had experienced bullying completed the entire survey).

After responding to questions about specific types of bullying, participants were then asked about their position at the time they were bullied, their response to bullying, the rationale for their responses, and the outcome of their actions. They were also given the opportunity to provide additional details in an open-ended format.

All participants were asked if they had ever witnessed academic bullying with someone else as the target. A similar series of questions was asked of witnesses.

Finally, all participants were asked a series of demographic questions such as their sex, ethnicity, current role, area of research, whether or not they were citizens of the country in which they studied or worked, and the country in which the bullying took place.

### 2.4 Complementary study on COVID-19 pandemic

This complementary survey was performed to obtain more data on the impact of COVID-19 pandemic on academic bullying. Data were collected from 191 individuals whose participation was solicited through various means including advertisements in *Science* magazine (through third-party emails) and the American Chemical Society (through their online panel advertisement and third-party emails). Participants were 17% junior faculty, 17% postdocs, 17% doctoral students, 2% undergraduate students, 2% visiting scholars, and 45% “Other” (indicating that staff members or other professionals were respondents on this survey). A majority of participants came from the fields of Biology (31.4%) and Chemistry (25.5%). Other fields were below 7.4%. Among our participants, 88% work or study in universities/colleges, and the majority were in their thirties (33.3%) or forties (23.2%). Fifty-three percent were U.S. citizens while 46% were not. Forty-nine percent work in US Universities; 49% did not.

### 2.5 Measures

Similar to our main study, after giving their informed consent to participate, respondents were given a definition of academic bullying. We then asked for information about the participants’ age, gender, position, and US citizenship status. They were then asked to indicate if they had ever been the target and/or witness of academic bullying. Those who answered “yes” were directed toward questions about differences in bullying behaviors before and after the pandemic.

### 2.6 Statistical analysis

To analyze the data, we used a variety of statistical techniques. When testing for significant statistical differences, we used either Chi-square or ANOVA. Chi-square analysis is appropriate when a variable is categorical (*e.g.*, male *vs.* female). ANOVA is appropriate when comparing differences among the means of two or more groups (*e.g.*, do male or female perpetrators score higher on the Tepper scale for abusive supervision?).

### 2.7 Ethic statement

The study has been reviewed and approved by IRB committees at Wake Forest University (IRB00023594) and Michigan State University [STUDY00003215 (for the main study) and STUDY00005250 for the complementary COVID-19 study)]. The consent forms were obtained from participants prior to filling out the survey questions. In the consent form, we provided full information about the study (including IRB information, the use of Qualtrics as the administrator of the survey, and approximate time needed to complete the survey). We indicated that the participation in this research was completely voluntary and the participants could discontinue their participation at any time without penalty by simply closing their browser window. The participants were informed that they had the choice not to answer any question(s) they did not wish to answer for any reason. We also informed the participants that while there would no particular benefits accrue to them, as a result of participating in this study, we believe that there would much to be gained in the field of academic science by understanding the extent to which bullying takes place in institutions of higher learning and science. We also informed the participant that they can address their questions or concerns about this study or the process of data collection, by contacting the co-principal investigators of this study (*i.e.*, the authors of this research) and/or institutional review boards. Full information on the consent and declaration of informed consent to use the data from the participants is available in the Appendix file.

### 2.8 Role of the funding source

There was no funding associated with this study. All authors had full access to all the data in the study and had final responsibility for the decision to submit for publication.

## 3 Results

**Fig. 1:**
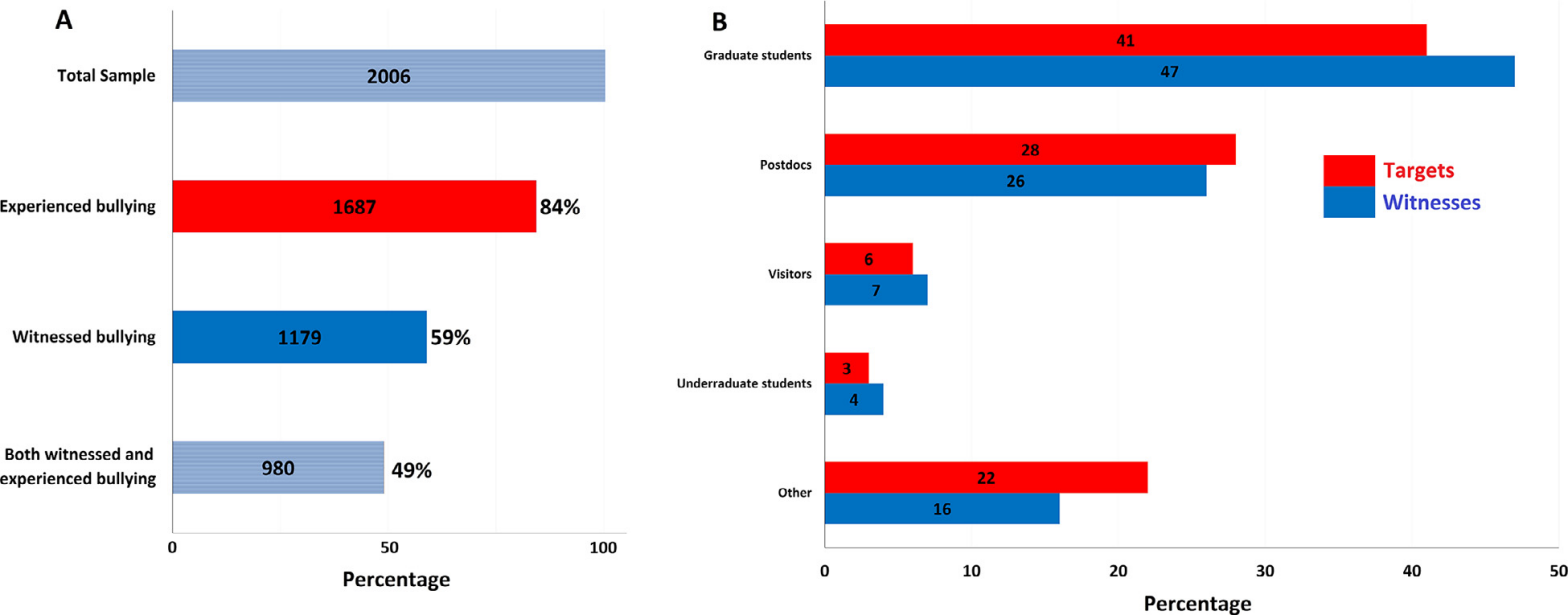
Information on participants, targets, and witnesses. (A) the total number of survey participants as well as the numbers of individuals who were targets, witnesses, and both targets and witnesses of academic bullying and (B) targets’ and witnesses’ reports of victim positions.

Before presenting the results of our hypothesis testing, we call the reader's attention to Fig. 1, which shows that of the total sample (*n* = 2006), 84% reported being the target of academic bullying, 59% reported being witnesses to bullying, and 49% reported being both targets of and witnesses to bullying. These results were based on the single item which provided a definition of academic bullying and asked participants if they'd been targeted or had witnessed bullying. While we directed our survey toward all individuals in academic science, it is very likely that targets and witnesses had more motivation to participate in the study than those with no experience with bullying. Despite this likely bias, we still find these percentages extraordinary, especially in comparison to other estimates of abusive supervision in non-academic organizational contexts, which hover around 10–14% [42]; or in academic contexts, typically 25–33% [6] but may be as high as 42% [16]. However, all remaining results should be interpreted under the assumption that our survey is very likely skewed, in that targets and witnesses were more likely to respond than those with no experience with academic bullying.

To address our research question, “Which bullying behaviors occur most frequently?” We administered the Tepper scale as well as the checklist of context-specific academic bullying behaviors developed for the current study. Using the Tepper scale for abusive supervision, the mean level of abusive supervision reported by targets was 3.23 on a scale of 1 (“I cannot remember him/her ever using this behavior with me”) to 5 (“He/she uses this behavior very often with me”). In most studies of abusive supervision, means are in the 1–1.5 range, but these studies include individuals who were not targeted. Note that in our survey, once participants indicated that they were neither targets of or nor witnesses to abuse, they were routed to the demographics section at the end of the survey. The top five abusive behaviors (see the full outcomes in Table 1) were (i) “makes negative comments about me to others (3.73); (ii) “does not give me credit for jobs requiring a lot of effort” (3.69); (iii) “breaks promises he/she makes,” (3.5); (iv) “lies to me,” (3.46); and (v) “puts me down in front of others” (3.43). Means range from 2.59 (“does not allow me to interact with my coworkers”) to 3.73.

The data we collected also provide support for the use of the checklist we developed to more precisely address bullying behaviors prevalent in the context of academic science. The top behaviors (see the full outcomes in Table 2) reported by targets were: “Encouraged others to mistreat me” (53.1%), “Canceled or threatened to cancel my current appointment/position” (52.1%), “Gave me a bad/unfair recommendation” (48.0%), “Took away my funding or threatened to take away my funding” (43.1%), and “Violated authorship contribution guidelines” (41.0%).

Hypothesis 1 suggested that perpetrators would be hierarchically superior to targets. Both targets and witnesses were most likely to report PIs as perpetrators (56.2% and 50.2%, respectively). The “other” category was chosen as the second most selected category for targets (23.2%) and “professor” was the second most selected category for witnesses (27.3%). We, however, acknowledge the possible role of discrepancy in the survey questions for targets and witnesses on this outcome. Open-ended responses from targets indicated that department chairs were perpetrators 15% of the time, senior faculty 12% of the time with deans, assistant deans, Ph.D advisors, and senior lab colleagues/peers between 6 and 8% of the time. It was not unusual for a target to report multiple perpetrators. We believe that the “other” category was chosen often due to differences in the nomenclature used to describe stakeholders in different fields and different countries.

At the same time, our results reveal that a majority of the targets of bullying were primarily graduate students (41%), post-docs (28%), with some visiting scholars (6%), undergraduate students (3%) and other (22%). Witnesses reported that 47% of targets were graduate students and 26% post-docs. Again, “other” was a category selected more than we expected, indicating that bullying extends beyond the PI-student relationship and may include other targets such as administrative assistants, junior colleagues, or possibly individuals in hierarchically superior positions. Overall, it appears that the majority of perpetrators (*e.g.*, PIs) were hierarchically superior to the majority of targets (*e.g.*, graduate students and Post Docs).

**Fig. 2:**
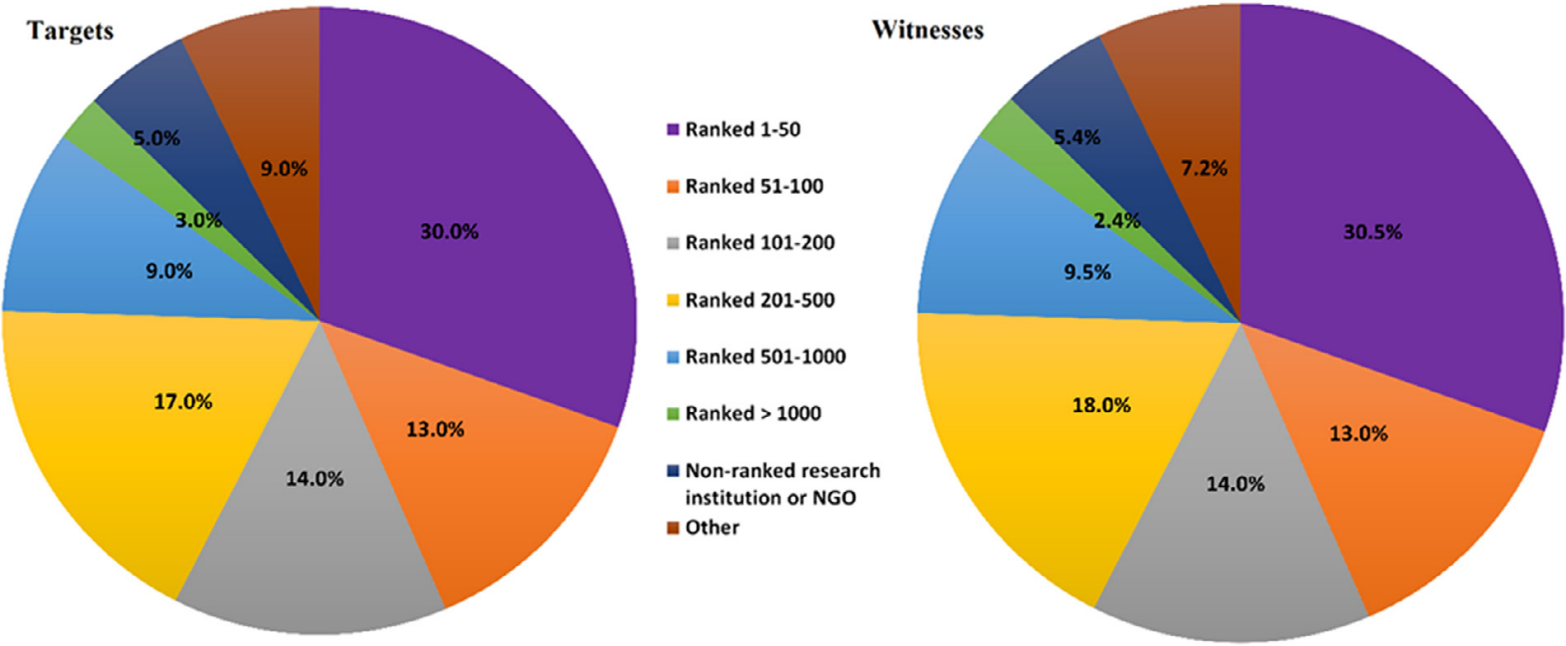
High-ranked universities are more prone to academic bullying behavior. Pie chart showing the percentage of institutional ranking where the bullying incidents took place according to the targets (*n* = 1151) and witnesses (*n* = 1010) reports.

Hypothesis 2 suggests that bullying is more likely in higher-ranked institutions. Fig. 2 shows the percentage of bullying according to the rank of their institutions. We provided a link (https://www.timeshighereducation.com/world-university-rankings/2019/world-ranking#!/page/0/length/25/sort_by/rank/sort_order/asc/cols/stats) in the survey which allowed participants to look up their institution's rank. Our data suggest that the largest percentage of abuse was reported in the highest ranked institutions. These data support the possibility that the highest percentages of abuse are reported from higher-ranked institutions.

Hypothesis 3 states that perpetrators are more likely to be male. We asked both targets and witnesses to indicate the gender of the perpetrator. Targets reported that males were the perpetrators 63% of the time, while witnesses reported male perpetrators 64% of the time. When we broke down the data set to include only self-reported targets and witnesses with STEM-related research areas (*n* = 718), both targets and witnesses reported that perpetrators were male 67% of the time. When we broke down our sample even further to include only targets in the US in STEM fields (*n* = 330), the percentage of male perpetrators was 63%. However, these statistics are difficult to interpret without context. A study conducted by the National Science Foundation (NSF, 2019 [43]) reports the percentage of females with doctoral degrees in several areas. Since 1997, the percentage of female PhDs in the physical sciences ranged from 13 to 19% and the percentage of women with PhDs in engineering ranged from 12 to 24%. Of the science and engineering doctoral degrees awarded in 2016, 41% were earned by women. From these statistics, we can conclude that women comprise a minority of STEM professionals with terminal degrees (in the US), suggesting that the proportion of male perpetrators of academic bullying (*i.e.*, 63–67%) reported by our respondents is roughly consistent with the proportion of men in these fields.

Before concluding that males are no more likely than females to be perpetrators of academic bullying, we ran two additional analyses. Going back to the full data set, we found that targets gave female perpetrators significantly higher scores on the 15-item Tepper scale (mean = 3.32) than male perpetrators (mean = 3.20) (*F* = 4.473, *p* < 035). This indicates that female perpetrators engage in a significantly higher frequency of abusive behaviors than male perpetrators, according to our participants. Another possibility may relate to the interaction of perpetrator and target sex, as studies have revealed that females will experience more indirect bullying from female supervisors and males will experience more direct bullying from male supervisors [44]. While not formally hypothesized, we conducted a 2 (perpetrator sex) x 2 (target sex) ANOVA to determine if there were any interactions between the sex of the perpetrator and of the targets on reports of abusive supervision. The effects were not significant (*F* = 0.038, *p* = 846).

We also ran a series of Chi-square analyses to determine if there were differences in the reporting of specific contextual behaviors from male and female perpetrators. While overall, there were no significant differences, we did find that targets reported that male perpetrators were trending in the direction of unnecessarily lengthening the target's stay (calendar time) in the lab (39 *vs.* 31%; χ^2^  = 2.786, *p* < 095) compared to female perpetrators. Based on all of these results, we conclude that males are no more likely to be perpetrators of bullying than females, but female perpetrators receive higher scores on the abusive supervision scale. Hypothesis 3 is not supported.

Hypothesis 4 predicted that targets of academic bullying are disproportionally women. Table 3 provides an overview of our results. As mentioned above, the majority (65%) of our participants were female, and 84% of participants reported being targets of academic bullying. To determine whether females were more likely than males to report being targets of academic bullying, we conducted a Chi-square analysis to compare the male/female proportion of the full sample (*n* = 2206) to those who reported being targets of abuse. Females were significantly more likely (87%) than males (78%) to report being targets of academic bullying (χ^2^ = 13.225, *p* < 004) based on the simple question, “Have you experienced academic bullying?”

**Table 3:** Results of Analysis of Differences Between Male and Female Targets of Bullying

Bullying Measure Used	Full Data Set Results	Global STEM Results	US STEM Results
1-item “Have you ever experienced (i.e., been the target of) academic bullying?	Females more likely to say yes	Females more likely to say yes	No significant difference

Tepper 15-item scale	No significant difference	No significant difference	No significant difference

Contextual checklist	Males more likely to experience 3 contextual behaviors	Males more likely to experience 3 contextual behaviors	Males more likely to experience 4 contextual behaviors

Next, we conducted subgroup analyses. We looked first at the Global STEM subgroup (removing social scientists and those not reporting sex, *n* = 837) and found that females in STEM (86%) were significantly more likely to report being targets of academic bullying than males (79%) (χ^2^ = 7.758, *p* < 0.005).

Finally, we analyzed the U.S. STEM-only subgroup (*n* = 428) and found that 86% of the males and 90% of females reported being bullied. However, this difference did not reach statistical significance (χ^2^ = 0.837, *p* = 0.344).

To further examine the relationship between bullying and target sex, we compared the mean level of abusive supervision on the Tepper scale and percentages of contextual bullying behaviors against male and female targets. Using the full data set (*n* = 2206), we found no differences between male and female targets’ reporting of abusive supervision on the Tepper scale (*F* = 0.152, *p* < 0.929). Using the STEM-only data set (*n* = 644), we found no significant differences on the Tepper abusive supervision scale between male and female targets (*F* = 0.117, *p* < 0.732) nor did we find significant differences on the Tepper scale between males and females (*F* = 0.652, *p* < 0.43) for the US-STEM only subgroup.

Next, we examined differences between male and female targets’ reporting on contextual bullying behaviors. When we examined the full data set, we found several significant differences. Male targets were more likely than female targets to report threats to their visas (12% *vs.* 7.6%; χ^2^ = 4.477, *p* < 0.034), threats to their funding (48% *vs.* 41%; χ^2^ = 3.987, *p* < 0.046), and authorship violations (46% *vs.* 38%; χ^2^ = 4.151, *p* < 0.042). When examining the Global STEM-only data set (*n* = 837), we found that males were more likely than females to report threats to their visas (13% *vs.* 8%) (χ^2^ = 4.06, *p* < 0.044), threats to their funding (49.5% *vs.* 41%) (χ^2^ = 4.206, *p* < 04), and were trending toward being more likely to experience authorship violations (48% *vs.* 40%) (χ^2^ = 3.654 *p* < 0.056).

When we examined the US STEM-only subgroup, we found that males were more likely than females to report having their funding threatened (53% *vs.* 41%; χ^2^ = 4.206, *p* < 0.040), and to have to sign away their rights (21% *vs.* 11%, χ^2^ = 5.959, *p* < 015). Our results also indicate that males were trending toward beingmore likely than females to report the threat of visa cancelation (19% *vs.* 11%) (χ^2^ = 3.179, *p* < 0.075) and to have their authorship rights violated (48% *vs.* 37%, χ^2^ = 3.732, *p* < 0.053).

This pattern of results suggests that women perhaps have a lower threshold for what they consider bullying and are therefore more likely to *perceive/report* being bullied (*e.g., via* a simple question such as “have you been bullied”?) while males were more likely to report experiencing a higher level of certain contextual bullying behaviors. There is significant consistency within measures of academic bullying across the different samples/sub-groups. Our data suggest mixed, and surprising results for Hypothesis 4.

Hypothesis 5 states that victims of academic bullying are disproportionately international. We asked participants in what country the bullying took place. Sixty countries were represented in the sample, with the largest percentages coming from the U.S. (47.9%), followed by the U.K. (11.2%), Germany (5.3%), and Canada (4.3%). To focus our analyses, we conducted a sub-group analysis using only the portion of the data from the U.S., including only targets of bullying with STEM research areas (*n* = 371) and found that 42.4% of the self-reported targets of bullying were not residents of the U.S. The majority of this group comprised graduate students (22.4%), post-docs (24.4%), junior faculty (16%), and “other” (24.2%). The more frequently reported research areas were life science (24.8%), engineering (13.6%), chemistry (12.7%), and neuroscience (9.7%). We then compared our data with publicly available data on the percentage of graduate students and post-docs in the U.S. in these areas. An Inside Higher-Ed study reports that the number of international students in various engineering fields ranges from 57 to 81%.[45] The percentage of international students in chemistry was reported to be 42.3% in 2008 by the American Chemical Society [46]. Another report demonstrates that 49% of STEM-educated scientists in the U.S. are foreign-born (National Science Board, 2020 [47]). This latter report suggests that the number of individuals studying in STEM programs in the U.S. has grown substantially between 2001 and 2017, with 46.2% of temporary visa holders entering graduate education in engineering, natural sciences, and social/behavioral sciences. Comparing these figures (range 42.3–81%) to the percentage of non-citizens reporting being bullied in our US STEM sample (41.1%), it appears that Hypothesis 5 is not supported. International students, post-docs, and early-career scholars are not any more likely than domestic students to *report* being targets of bullying, at least not in the U.S.

However, the previous finding pertains only to the question, “Have you been a target of academic bullying?” We conducted some post-hoc analyses on this same sub-group (US STEM) to determine whether international scholars reported higher frequencies of abusive behaviors than domestic scholar participants. We first examined their scores on the Tepper scale and found that there was no significant difference (*F* = 0.686, *p* = 0.408) between domestic and international scholars. However, when we examined differences on the contextual items, a higher percentage of international scholars naturally reported more threats of visa cancelation (32% *vs.* ∼0%; χ^2^ = 65.28, *p* < 0.0001), violations of intellectual property rights (30% *vs.* 20%; χ^2^ = 4.163, *p* < 0.041), and threats of position cancelation (60% *vs.* 46%; χ^2^ = 6.189, *p* < 0.013) than domestic scholars. The data also trends toward international scholars being more likely to have their data used without acknowledgement (43% *vs.* 32%; χ^2^ = 3.718, *p* < 0.054). Thus, we can conclude that while international scholars may not be disproportionately targeted, when they *are* targeted, the *severity* of certain contextual abuses is higher. Our findings provide substantial support for Hypothesis 5.

Hypothesis 6 suggested that targets of bullying and witnesses would be more likely to use “flight” *vs.* “fight” tactics in response to bullying. Over 64% of targets reported “flight” responses (*i.e.*, 27% did not report the bullying and 37% sought emotional support). Only 29% reported abuse to their institutions. Witnesses reported that “flight” responses were chosen by targets 78% of the time (*i.e.,* 46% didn't report and 32% sought support from friends/colleagues). Only 16.5% of witnesses said that the targets reported the bullying to their institutions. Further, witnesses also chose non-confrontational responses 85.0% of the time, including not reporting (25.3%) but offering support to the victim (59.5%). Only 11% of witnesses reported the bullying to their institutions. These data support the idea that targets and witnesses are more likely to use “flight” tactics as a response to bullying.

Hypothesis 7 suggested that fear of retaliation would be the primary reason for use of avoidant, non-confrontational tactics. Indeed, both targets and witnesses reported fear of retaliation as the primary reason for failure to report (61% and 62.4%, respectively). While there is support for Hypothesis 7, a significant proportion of targets chose the “other” category as their reason for not reporting. Qualitative analyses of open-ended responses (*n* = 723; Appendix 1 of the Supplementary Information) indicated that participants were eager to share additional details of their experiences with bullying. We coded the responses of the 723 responding to the open-ended item, 122 of which mentioned their reason for not reporting the bullying. Some reported more than one reason for not “fighting” but overwhelmingly, 87% elaborated on the “fear of retaliation” theme. Smaller proportions mentioned fear of losing visa or that they were unaware of resources available to them. Table 4 provides sample responses.

**Table 4:** Samples of qualitative comments in open-ended survey question (rationale).

Fear of Retaliation	Fear of Visa Cancellation	Lack of Informational Resources
*Since this person is in power positions not only in university but also in National Research Council, I decided not to report because it can threaten my future career in science. These fears are based on true stories of other scientists who had a conflict with this person because of the same reason, and their careers were affected severely.*	*Was informed I would most likely lose my job and my visa relied on my job.* *I was in a dependent situation due to VISA status.*	*Didn't know who I could complain to or what the outcome would be. Couldn't imagine any positive outcomes from reporting.* *There was nobody to complain to. I asked my advisor from my PhD if there was anything that I could do about my treatment during my postdoc and he said no. I decided not to complain publicly.*

87%	6.5%	12%

Hypothesis 8 proposed that institutional responses to targets’ reports of bullying would be inadequate. Targets reported that outcomes were “unfair and biased” 58% of the time, and “fair and unbiased” only 8% of the time. A significant percentage (34%) of the targets selected “other” as the outcome, so we conducted a qualitative analysis of their open-ended responses (*n* = 723; Appendix 1 of the Supplementary Information) and found, again, that participants were not reluctant to share details of their experiences. Of the 723 who responded to the open-ended questions asking for details of their experience as a target of bullying, 388 mentioned the outcomes. When analyzing the narratives they provided, we found that 41% reported that nothing happened following their report; 34% elaborated on the retaliation theme; 25% left the lab, institution or field; 16% reported that the bully was protected; and only 13% reported being supported by the institution. Table 5 provides sample responses below.

**Table 5:** Samples of qualitative responses to open-ended survey question (outcomes)

Retaliation	Bully Protected	Left Lab/Institution/Field	Target was Supported	Nothing Happened
*I first spoke up, but this made the situation worse. Then, I reported to higher level people in my department and then to dean's office. They destroyed my life and my scientific identity as well as my dignity. They crushed my entire career. Yes, I got a lot of retaliation.*	*I complained; although the investigation committee validated my allegations, they did nothing to my supervisor. I was the one who had to leave, because they asked me to continue working under my supervisor and report if additional incidences happened!*	*Very famous professor in my field, with a university structure without a fair ombudsman system (i.e., no one would want to support me against the professor due to his high rank and prestige). Although I spoke to colleagues about the situation, it was generally seen as an unavoidable situation and something to just accept. Situation became so bad that I quit my PhD and changed fields to avoid further interaction with the professor.*	*I talked to the Ombudsman and the Dean who both supported me and further talked to the head of the XXX so that my appointment wasn't cancelled. It was cut short but not as much as initially threatened. I got therapy hours from the institute to help cope, 10 hours, and meetings with the ombudsman to keep contact and let me know they hadn't forgotten about me.*	*I spoke to multiple PIs and everyone was aware of the situation (i.e. that the person abused his staff, wife and children). Nobody dared to intervene as he is a superstar scientist and also has his nice and kind side when he is not stressed.* *Spoke to department chair and was told I am the problem; Spoke with ombudsperson and was told I am NOT the problem but because it was not gender based bullying, there was nothing that could be done.*

34%	16%	25%	13%	41%

Further, witnesses reported 54% of the time that the outcomes of reporting were unfair and biased while only 7% reported fair and unbiased results (*n* = 723; Appendix 1 of the Supplementary Information). There is substantial evidence to support Hypothesis 8.

Finally, we wanted to know if the COVID-19 pandemic has had any impact on the experience of academic bullying. Most of the data for the main study were collected before the pandemic, but in September 2020, we added an additional item to the end of the survey, asking those who had either experienced or witnessed bullying, if and how COVID-19 had affected bullying behaviors (exacerbated, no effect or reduced). According to the outcomes of the main study, where 206 participants responded to the COVID question, 45.6% said bullying was exacerbated by COVID-19, 40.3% said COVID-19 had no effect on bullying, and 13.1% said COVID-19 reduced bullying.

To obtain more clarity on the impact of the pandemic on bullying behavior, we conducted a separate, complementary survey (see the Methods section for details). A total of 191 participants provided responses. In this survey, we asked two main questions regarding the frequency and pattern of academic bullying: (1) Had participants either witnessed or experienced bullying before the pandemic and during the pandemic; and (2) If witnessed/experienced, were the bullying behaviors exacerbated, reduced or the same. Table 6 indicates that the frequency of bullying had decreased during the pandemic. However, 39% of participants reported that the severity of the bullying had gotten worse (49% reported no change and 12% reported reduced severity). We suspect that the reduced frequency is likely due to social distancing measures instituted in labs and other workplaces. We suspect that increased severity is the product of greater pressure experienced by all affected by the pandemic. Thus, we conclude that bullying was less frequent during the pandemic but with a higher level of severity.

**Table 6:** Effect of COVID-19 pandemic on the *frequency* of bullying behavior.

	Before pandemic	During pandemic
Experienced	21%	17.6%
Witnessed	17.4%	12.8%
Experienced & Witnessed	35.8%	21.9%
Neither	25.8%	47.6%

*n* = 191.

## 4 Discussion

While bullying in academia has been acknowledged, there have been relatively few empirical investigations of the phenomenon. In this study, we attempted to elucidate the forms of abuse, the most likely perpetrators and targets, as well as the typical reactions of targets and witnesses. Finally, we investigated the results of targets’ actions following chronic bullying. We received responses from all over the globe, likely due to growing concern regarding this issue in the science world and several high-profile cases at prestigious institutions [23,34,48,49]

Since we defined academic bullying as “sustained hostile behavior from one's academic superior” we were not surprised that a majority of perpetrators were PIs and others who were hierarchically superior to the typical targets, who were mostly graduate students and postdocs. These results underscore the importance of power differentials as important antecedents of academic bullying, suggesting that PIs and other organizational leaders may need training on supportive leadership behaviors before being granted their own labs or leadership positions.

We found that while males make up a majority of perpetrators, they do not disproportionally bully their subordinates. They are simply the majority in positions of power in most STEM fields and are proportionately represented among perpetrators. However, high-profile cases such as that of Nazneen Rahman [50] reveal that the face of academic bullying has no gender. In fact, our data show that the targets of female perpetrators reported higher levels of abusive supervision behaviors on the Tepper scale than did targets of male perpetrators.

Females make up a majority of those *reporting* that they have been bullied, in the full data set and in the Global STEM subgroup. However, the specifics of our analysis shed additional light on nuances in the relationship between perceived bullying and target gender. When asked if they had been targets of bullying, females were more likely to say yes. However, when reporting on the experience of specific behaviors in the Tepper scale, there were no significant differences between the male and female targets. When we examined the differences in the contextual behavior checklist, there were a number of significant differences, with male targets reporting worse treatment. Further, detailed research regarding the role of gender in all aspects of academic bullying in the STEM fields is required before substantive conclusions are possible.

“Otherness” and increased vulnerability due to visa issues, particularly for foreign graduate students and postdocs (the most frequent targets), increases the severity of contextual bullying and their patterns in the US. As more international students enter STEM fields, this may increase both the severity of bullying overall and the probability that these behaviors will trickle down to future scientists. With the percentages of international graduate students and postdocs in the US increasing, the increased severity of bullying towards international scholars underscores the urgent need for interventions from institutions, funding agencies, and individual scientists to address these behaviors from all angles [15,[51], [52], [53]].

This urgent need is even more evident when we consider that more bullying seems to take place in the most prestigious institutions. Thousands of graduate students and postdocs apply for positions working with famous scientists in the world's most highly regarded academic institutions. This creates a powerful breeding ground for bullying, because perpetrators have even more leverage as targeted students justify suffering abuse in exchange for prestige. If they choose to leave, they can be easily replaced by eager applicants from all over the world. While the power inequalities between PIs and graduate students/postdocs in any institution are already obvious, especially in STEM, it is exponentially greater in the highest-ranked institutions, leaving these institutions even more exposed. We are encouraged by the work under way in several institutions, including Duke University [54], the University of Wisconsin Madison [55], and the University of California (through establishment of the National Center for Free Speech and Civic Engagement) [56] to shed light on this important issue, not only increasing awareness but actually cultivating policies and practices designed to curb bullying.

Despite these bright spots, the results of our study indicate that much work remains to be done. When targets and witnesses choose not to report bullying because they fear retaliation or when they do, find that the results of speaking out are unfair and biased, as our data clearly suggests, the field has not evolved far enough. Much more needs to be done to develop policies and procedures, akin to those pertaining to sexual harassment, to protect the rights of junior/new, budding scientists, or their fields may be robbed of the scientific findings that hugely affects scientific integrity and breakthrough progress.

Last but not least, our results suggest that the current COVID-19 pandemic is having a significant effect on the frequency and pattern of academic bullying. While the frequency of bullying reported by targets and witnesses has decreased, likely due to social distancing, the severity has increased, likely due to increased pressures on everyone due to the pandemic. Institutions must be sure to focus their attention on this matter during this difficult time.

Finally, we acknowledge the main limitation of our study, which is the fact that we limited our focus on bullying to those in hierarchically higher positions, as our definition directed participants toward “academic superiors.” Thus, there is still a need to focus empirical attention on varying types of bullying from colleagues or others in the academic workplace, including mobbing [16]. We also acknowledge the probability that those who had either witnessed or experienced bullying were more likely to complete the survey than those who hadn't, creating a sampling bias. We also acknowledge that a large percentage of our data comes from the United States. Finally, we note that the design of our survey may have caused us to miss out on some interesting results. When participants initially indicated that they had not experienced or witnessed academic bullying, they were routed to other parts of the survey and did not respond to the Tepper abusive supervision scale nor the contextual checklist or the institutional ranking item. Perhaps some of these participants would have indicated that they had indeed experienced or witnessed some of abusive behaviors our survey addressed. Further, this design issue may have skewed our results regarding institutional ranking as we do not know how many respondents who *were not* targets of bullying came from top, middle or bottom-ranked institutions. Despite these potential biases to the generalizability of our results, we have attempted to illuminate, in greater detail, the types of behaviors most likely experienced by targets of bullying, as well as their typical responses and resulting outcomes.

In summary, our empirical investigation of academic bullying has illuminated some of the less-familiar patterns and nuances of bullying behaviors in academic science. We hope that our results will serve to rally all stakeholders, especially those in a position to make a difference in creating a safe and positive environment for scientists and budding scientists around the world.

## Declaration of Competing Interest

Morteza Mahmoudi discloses that he is a co-founder and director of the Academic Parity Movement (www.paritymovement.org), a non-profit organization dedicated to addressing academic discrimination, violence, and incivility. Sherry Moss discloses that she is a director of the Academic Parity Movement.

## References

[1] Mahmoudi M., Keashly L. (2021). Filling the space: a framework for coordinated global actions to diminish academic bullying. Angew Chem.

[2] Kowalski R.M., Limber S.P. (2013). Psychological, physical, and academic correlates of cyberbullying and traditional bullying. J Adolesc Health.

[3] Prevost C., Hunt E. (2018). Bullying and mobbing in academe: a literature. Eur Sci J.

[4] Smith D.K. (2020). The race to the bottom and the route to the top. Nat Chem.

[5] Iwasaki A. (2020). Antidote to toxic principal investigators. Nat Med.

[6] Keashly L. (2021). Special topics and particular occupations, professions and sectors.

[7] Tepper B.J. (2000). Consequences of abusive supervision. Acad Manag J.

[8] Tepper B.J., Simon L., Park H.M. (2017). Abusive supervision. Annu Rev Organ Psychol Organ Behav.

[9] Tepper B.J., Moss S.E., Duffy M.K. (2011). Predictors of abusive supervision: supervisor perceptions of deep-level dissimilarity, relationship conflict, and subordinate performance. Acad Manag J.

[10] Lyu Y., Zhou X., Li W., Wan J., Zhang J., Qiu C. (2016). The impact of abusive supervision on service employees’ proactive customer service performance in the hotel industry. Int J Contemp Hosp Manag.

[11] Pan W., Sun L., Sun L.Y., Li C., Leung A.S. (2018). Abusive supervision and job-oriented constructive deviance in the hotel industry. Int J Contemp Hosp Manag.

[12] Zhao H., Guo L. (2019). Abusive supervision and hospitality employees’ helping behaviors. Int J Contemp Hosp Manag.

[13] Iwasaki A. (2020). Antidote to toxic principal investigators. Nat Med.

[14] Bullying of PhDs (1998). Nature.

[15] Mahmoudi M., Keashly L. (2021). Filling the space: a framework for coordinated global actions to diminish academic bullying. Angew Chem.

[16] Keashly L., Neuman J.H. (2010). Faculty experiences with bullying in higher education: causes, consequences, and management. Adm Theory Prax.

[17] Garcia P.R.J.M., Restubog S.L.D., Kiewitz C., Scott K.L., Tang R.L. (2014). Roots run deep: investigating psychological mechanisms between history of family aggression and abusive supervision. J Appl Psychol.

[18] Mawritz M.B., Mayer D.M., Hoobler J.M., Wayne S.J., Marinova S.V. (2012). A trickle-down model of abusive supervision. Pers Psychol.

[19] S. Farley, C. Sprigg Culture of cruelty: why bullying thrives in higher education. Retrieved April 2014; 7: 2015.

[20] Nelson E., Lambert R. (2001). Sticks, stones and semantics: the ivory tower bully's vocabulary of motives. Qual Sociol.

[21] Mahmoudi M., Moss S.E. (2019). Tie institutions' reputations to their anti-bullying record. Nature.

[22] Jordão E.M.A. (2019). PhDs in Brazil are perishing even when they publish. Nat Hum Behav.

[23] Else H. (2018). Does science have a bullying problem. Nature.

[24] Waldman D.A., Wang D., Hannah S.T., Owens B.P., Balthazard P.A. (2018). Psychological and neurological predictors of abusive supervision. Pers Psychol.

[25] Kiazad K., Restubog S.L.D., Zagenczyk T.J., Kiewitz C., Tang R.L. (2010). In pursuit of power: the role of authoritarian leadership in the relationship between supervisors’ Machiavellianism and subordinates’ perceptions of abusive supervisory behavior. J Res Pers.

[26] Hobfoll S.E. (1989). Conservation of resources: a new attempt at conceptualizing stress. Am Psychol.

[27] Witze A. (2018). Sexual harassment is rife in the sciences, finds landmark US study. Nature.

[28] Mahmoudi M., Moss S. (2019). Scarcity of lab positions in high-ranked institutions creates a breeding ground for bullies. BioImpacts BI.

[29] http://uis.unesco.org/sites/default/files/documents/fs51-women-in-science-2018-en.pdf.

[30] Van den Brink M., Stobbe L. (2014). The support paradox: overcoming dilemmas in gender equality programs. Scand J Manag.

[31] Buse K., Bilimoria D., Perelli S. (2013). Why they stay: women persisting in US engineering careers. Career Dev Int.

[32] Gold J.A., Roubinov D., Jia L.S. (2020). Gender differences in endowed chairs in medicine at top schools. JAMA Intern Med.

[33] Ma Y., Oliveira D.F., Woodruff T.K., Uzzi B. (2019).

[34] Abbott A. (2019). Germany's prestigious max planck society conducts huge bullying survey. Nature.

[35] Opotow S. (1990).

[36] W.B. Cannon The wisdom of the body. 1939.

[37] H. Rachlin Behavior and learning. 1976.

[38] Hess M., Bullying S.H. (2015).

[39] Tepper B.J., Moss S.E., Lockhart D.E., Carr J.C. (2007). Abusive supervision, upward maintenance communication, and subordinates' psychological distress. Acad Manag J.

[40] Von Elm E., Altman D.G., Egger M., Pocock S.J., Gøtzsche P.C., Vandenbroucke J.P. (2007). The strengthening the reporting of observational studies in epidemiology (STROBE) statement: guidelines for reporting observational studies. Bull. World Health Organ..

[41] https://www.sciencemag.org/features/2020/01/academic-bullying-desperate-data-and-solutions.

[42] A.C. Schat, M.R. Frone, E.K. Kelloway Prevalence of Workplace aggression in the US workforce: findings from a national study. 2006.

[43] https://ncses.nsf.gov/pubs/nsf19304/data.

[44] Vaillancourt T. (2013). Do human females use indirect aggression as an intrasexual competition strategy?. Philos Trans R Soc B Biol Sci.

[45] E. Redden Foreign students and graduate STEM enrollment: inside higher education 2017.

[46] https://www.acs.org/content/acs/en/education/students/graduate/survey-of-phd-programs-in-chemistry.html.

[47] https://www.nsf.gov/nsb/sei/one-pagers/Foreign-Born.pdf.

[48] Abbott A. (2018). Max Planck astrophysicist at centre of bullying allegations speaks up. Nature.

[49] Lewis D. (2019). Head of prestigious ancient-DNA lab suspended amid bullying allegations. Nature.

[50] Else H. (2018). Top geneticist loses pounds sterling 3.5-million grant in first test of landmark bullying policy. Nature.

[51] Mahmoudi M., Ameli S., Moss S. (2020). The urgent need for modification of scientific ranking indexes to facilitate scientific progress and diminish academic bullying. BioImpacts BI.

[52] Mahmoudi M., Moss S. (2019). Tie institutions’ reputations to their anti-bullying record. Nature.

[53] S. Moss Research is set up for bullies to thrive. Nature 2018; 560(7719): 529–30. 10.1038/d41586-018-06040-w 30154542

[54] https://hr.duke.edu/policies/diversity/harassment-discrimination.

[55] https://hr.wisc.edu/hib/.

[56] https://freespeechcenter.universityofcalifornia.edu/.

